# Comprehensive Evaluation of Three Important Herbs for Kombucha Fermentation

**DOI:** 10.17113/ftb.61.01.23.7789

**Published:** 2023-03

**Authors:** Burcu Emine Tefon Öztürk, Berfin Eroğlu, Eda Delik, Mustafa Çiçek, Esra Çiçek

**Affiliations:** 1 Biology Department, Faculty of Science, Akdeniz University, Dumlupınar Boulevard, 07058 Antalya, Turkey; 2 Department of Biology, K.Ö. Science Faculty, Karamanoğlu Mehmetbey University, İbrahim Öktem Boulevard, 70100 Karaman, Turkey; 3 Department of Biological Sciences, Middle East Technical University, Dumlupınar Boulevard, 06800 Ankara, Turkey

**Keywords:** kombucha, bioactivity, antibacterial activity, cytotoxic activity, sensory evaluation, phenolic substance

## Abstract

**Research background:**

Kombucha is consumed worldwide for its beneficial health effects. Kombucha teas fermented with various herbal infusions have become very important nowadays. Although black tea is used for kombucha fermentation, kombucha teas fermented with different herbal infusions have gained great importance. In this study, three different traditional medicinal plants, namely hop (*Humulus lupulus* L.), madimak (*Polygonum cognatum*) and hawthorn (*Crataegus monogyna*) were used for the fermentation of kombucha beverages, and the bioactivity of these beverages was investigated extensively.

**Experimental approach:**

The microbiological profile, bacterial cellulose formation, antibacterial, antiproliferative and antioxidant activities, sensory properties, total phenolic content and flavonoid content of kombucha beverages were investigated. Liquid chromatography-coupled mass spectrometry analysis was used to identify and quantify specific polyphenolic compounds in the samples.

**Results and conclusions:**

According to the results, the hawthorn-flavoured kombucha, which has lower free radical scavenging activity than the other samples, came into prominence in terms of sensory properties. All examined kombucha beverages showed a strong cytotoxic effect on Mahlavu and HCT116 cell lines, but only the madimak-flavoured kombucha sample, which had a higher total phenolic/flavonoid content, had antibacterial activity against all microorganisms used in the study.

**Novelty and scientific contribution:**

Considering the results of this study, madimak could be an effective herb for the development of new kombucha beverages, although its sensory properties still need to be improved. This study contributes to science in terms of producing new fermented beverages with improved beneficial health effects.

## INTRODUCTION

Kombucha is a slightly sweet and acidic beverage originating in Manchuria. It is traditionally prepared through fermentation of *Camellia sinensis* (black tea) and sugar by a symbiotic culture of acetic acid bacteria (mainly *Komagataeibacter*, *Acetobacter* and *Gluconobacter*) and osmophilic yeasts (*1*). *Komagataeibacter xylinus*, in particular, is regarded as the most prominent microorganism of kombucha fermentation (*2*). In the production of kombucha, the medium is usually inoculated with a biofilm of bacteria and cellulose pellicles formed during previous cultivation (*i.e.* symbiotic culture of bacteria and yeast or SCOBY) and then incubated for 7 to 14 days under aerobic conditions. Sucrose, the primary carbon source for kombucha fermentation, is eventually oxidized to acetic acid by acetic acid bacteria during fermentation. The beverage is reported to be effective against metabolic diseases, psoriasis, constipation, indigestion and hypertension (*3*). It is believed that the chemicals formed by metabolic activity such as lactic, acetic, glucuronic, and gluconic acids, free radical-binding vitamins such as C, B_2_ and B_6_, amino acids, antibiotics, catechins, and various micronutrients contained in the fermented tea are responsible for the beneficial properties of kombucha (*4*).

Since this fermented beverage is widely consumed due to its probiotic content, improving the antioxidant and antimicrobial properties of kombucha is an important issue. This situation prompted researchers to search for different aromatic plants and fermentation media rich in antioxidants, vitamins, nutrients and antibacterial substances (*5*). Numerous other studies have previously investigated the use of different herbs in kombucha preparation and evaluated their antioxidant and antimicrobial activities (*6*-*8*).

Hop (*Humulus lupulus* L.), madimak (*Polygonum cognatum*) and hawthorn (*Crataegus monogyna*) are herbs that have been used in traditional medicine for a long time. Hop has drawn a lot of attention in recent years for its sedative effects, digestive benefits, estrogenic properties and potential cancer-preventive effects. The antibacterial effects of hop on mainly Gram-negative bacteria have been well documented by a variety of research groups (*9*, *10*). Madimak, a medicinal plant, is used in traditional Turkish medicine to treat diabetes mellitus and urinary tract diseases (*11*). Hawthorn, the third herb used in the study, is a member of genus *Crataegus*. Plants of this genus have been reported to have cardioprotective, anticarcinogenic, antioxidant and anti-inflammatory effects on human health (*12*).

This study aims to investigate the antibacterial effect, microbiological profile, antioxidant and cytotoxic activities, total flavonoid and phenolic content, sensorial properties and cellulose production of kombucha teas fermented with medicinal plant infusions. Some polyphenolic compounds of the samples were identified and quantified by liquid chromatography-coupled mass spectrometry analysis (LC-MS/MS). The traditional medicinal plants used in this study, namely hop, madimak and hawthorn were selected for their therapeutic effects.

## MATERIALS AND METHODS

### Fermentation conditions of the kombucha beverages

In this study, black tea leaves (*Camellia sinensis*, Lipton, Unilever, Istanbul, Turkey), hop (*Humulus lupulus* L.) and hawthorn (*Crataegus monogyna*), both acquired from the local market in Kepez, Antalya, Turkey, and madimak (*Polygonum cognatum*), acquired from the local market in Karacaören, Sivas, Turkey, were used to prepare kombucha beverages. The kombucha used for the experiments was a local isolate (Antalya, Turkey). Traditional kombucha fermented with medicinal herbs were labelled hawthorn-flavoured kombucha (KHa), hop-flavoured kombucha (KH) and madimak-flavoured kombucha (KM). Infusion prepared using only black tea was labelled black tea (B) and those with only herbs were called herbal tea: hawthorn (Ha), hop (H) and madimak (M), and they were used as control groups.

The protocol defined by Marsh *et al*. (*13*) was adopted with minor modifications to prepare the traditional kombucha. Briefly, 5% dry black tea leaves (*m*/*V*) were steeped in 1 L of boiling water for 3 min. Then, 9% sucrose (Torku, Konya, Turkey) (*m*/*V*) was added, and the solution was boiled for another 1 min. Subsequently, the solution was cooled to room temperature, and the dry leaves were removed by filtration. A volume of 100 mL of this black tea infusion with 2% (*m*/*V*) SCOBY and *φ*=10% soup (starter culture) from the previous culture was inoculated into each glass jar for kombucha tea fermentation. The sample flasks were incubated at room temperature. After 14 days of incubation, the liquid portion of the kombucha beverages was collected and centrifuged at 1370×*g* (Mistral 2000; MSE, London, UK) for 10 min for further experiments.

To prepare herb-flavoured kombucha beverages, 5% dry black tea leaves (*m*/*V*) and 5% dry herbal leaves (*m*/*V*) were added together to 1 L boiling water containing 9% sucrose (*m*/*V*), and the same procedure described above was followed.

For herbal teas, 5 g of black tea, hop, madimak and hawthorn leaves were added to 1 L of boiled water containing 9% sucrose.

### pH measurement

The pH values of the samples were measured on days 0 and 14 using a pH meter (model 616.12.001; Isolab, Wertheim, Germany).

### Microbiological profile

Microbiological profiling of the samples was performed on days 0 and 14 of fermentation. A volume of 1 mL of samples was taken homogeneously from the fermentation vessels and serial dilutions of the samples were prepared with 0.05 M NaCl (Sigma Aldrich, Merck, St. Louis, MO, USA). Selective media were inoculated with 200 µL of the 10^-5^ and 10^-6^ diluted samples, and this step was repeated three times for each sample. In order to count yeasts, yeast extract glucose chloramphenicol (YGC) agar (Merck, Darmstadt, Germany) was used as described by Coton *et al*. (*14*). Glucose yeast extract calcium carbonate (GYC) agar was used for counting acetic acid bacteria (*15*). Plate count agar (PCA; Merck, Darmstadt, Germany) was used to determine mesophilic bacterial colonies. De Man Rogasa Sharp (MRS) agar (Merck) was used to count *Lactobacillus* (*14*). The colonies were counted and colony forming units (CFU) per millilitre were calculated after incubation for five days at 30 °C for MRS, PCA, and GYC plates and at 25 °C for YGC plates according to the following equation:


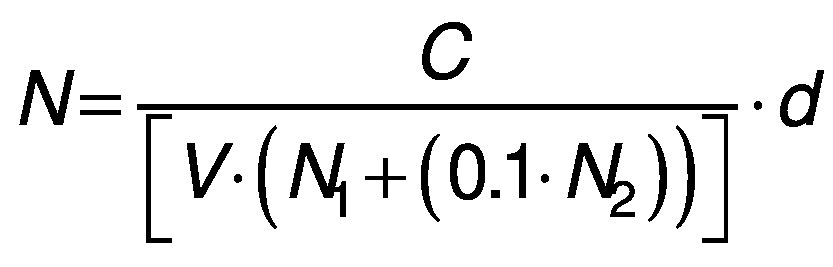
 /1/

where *N* is the total amount of microorganisms in one millilitre, *C* is the total number of colonies counted in a sample, *V* is the volume of serial dilutions transferred to the samples (mL), *N*_1_ is the number of replicates of samples prepared with the first of the serial dilutions, *N*_2_ is the number of replicates of samples prepared with the second of the serial dilutions, and *d* is the most concentrated of the successive serial dilutions (*16*).

### Measurement of cellulose pellicle production

In order to measure the dry mass of the pellicles, the cellulosic biofilm of the kombucha samples was washed with dH_2_O and incubated at 90 °C in 1% NaOH (Sigma Aldrich, Merck) for 15 min. After incubation, the samples were washed with dH_2_O and then treated with 1% glacial acetic acid (Merck) and air-dried (*17*).

### Determination of total phenolic content by Folin-Ciocalteu assay

The colorimetric method described by Škerget *et al*. (*18*) was used to identify the total phenolic content (TPC) of kombucha samples. The Folin-Ciocalteu (FC) reagent was prepared according to Singleton and Rossi (*19*). The soup of kombucha samples was collected and centrifuged at 1370×*g* (Mistral 2000; MSE) for 10 min. The supernatants were then filtered through a sterile filter with a pore diameter of 0.45 µm (GVS Filter Technology, Indianapolis, IN, USA). A volume of 500 µL of the filtered kombucha samples and 2.5 mL FC reagent (1:10) were mixed, vortexed and incubated for 2 min. Then, 2 mL of 7.5% Na_2_CO_3_ (Merck) were added to the samples, vortexed for 30 s and kept in a water bath at 50 °C for 5 min. TPC was expressed as gallic acid equivalent (GAE) in µg/mL after the absorbance values were measured at 760 nm with spectrophotometer (UV-5100; SOIF, Shanghai, PR China).

### Determination of total flavonoid content by aluminium chloride colorimetric method

The colorimetric method with aluminium chloride was used to identify the total flavonoid content (TFC) of kombucha samples (*20*). The soup of kombucha samples was collected and centrifuged at 1370×*g* (Mistral 2000, MSE, UK) for 10 min. Then, supernatants were filtered through a sterile filter with a pore diameter of 0.45 µm (GVS Filter Technology). To each of the 500 µL kombucha sample, 1.5 mL methanol (Isolab), 100 µL 10% AlCl_3_ (Merck), 100 µL 1 M potassium acetate (Merck) and 2.8 mL distilled water were added. Then, the solution was incubated for 30 min at 25 °C in the dark. Absorbance values were measured at 415 nm, and TFC was expressed as quercetin equivalents (QE) in µg/mL.

### Determination of phenolic compounds by LC-MS analysis

LC-MS analysis was performed with the Agilent HPLC 6430 system (Waldbronn, Germany) equipped with a C18 column (particle size 1.8 μm, 2.1 mm×150 mm i.d.) using the method developed by Fischer *et al.* (*21*). The resolution peaks were recorded on the HPLC chart according to the retention times of the standards prepared with methanol (Isolab). After dilution, each sample was homogenized with a vortex and centrifuged (Mistral 2000; MSE) at 3350×*g* for 10 min. The clear portion of the samples was collected and passed through 0.45 µm membrane filters (GVS Filter Technology) and injected into the LC-MS/MS instrument.

The study was performed in positive and negative ion modes. The mobile phase consisted of *φ*(formic acid)=0.01% (Merck Millipore, Darmstadt, Germany), 5 μM ammonium formate in methanol (Merck Millipore) and water (Merck Millipore) *φ*(methanol,water)=5% (eluent A) and *φ*(formic acid)=0.01% and 5 μM ammonium formate in methanol (eluent B). The flow rate was 0.3 mL/min, and the gradient program was optimized as follows: 5% B isocratic (1 min), 30% B (2 min), 60% B (1 min), 60% B isocratic (1 min), 70% B (1 min), 80% B isocratic (2 min), 100% B (2 min), 5% B isocratic (2 min) and 5% B isocratic (5 min). The injection volume of all samples was 3 μL and the total run time was 15 min.

### Measurement of free radical scavenging activity by DPPH method

The 2,2-diphenyl-1-picrylhydrazyl (DPPH) method described by Von Gadow *et al*. (*22*) was used to measure free radical scavenging activity (RSA) of the beverages. Kombucha samples were centrifuged at 1370×*g* for 10 min (Mistral 2000; MSE) and filtered through a filter with a pore diameter of 0.45 µm (GVS Filter Technology). A volume of 4 mL of methanolic DPPH solution (Sigma Aldrich, Merck) was added to 100 µL of the prepared samples and the reaction mixture was kept in the dark for 30 min. The absorbance of the samples was measured with a spectrophotometer (UV-5100; SOIF, Shanghai, PR China) at 516 nm and the results were expressed in ascorbic acid equivalents (AAE) in µmol/mL.

### Determination of antibacterial activity

The disk diffusion method was used to determine the antibacterial activity of the fermented beverages (*23*). *Bacillus cereus* (DSM 22648), *Escherichia coli* (ATCC 35218), *Klebsiella pneumoniae* (ATCC 13883), *Pseudomonas aeruginosa* (ATCC 27853), *Staphylococcus aureus* (ATCC 29213) and *S. epidermidis* (ATCC 12228) were the bacterial strains used in this study.

Fresh bacterial solutions were adjusted to 0.5 McFarland in NaCl (0.85% (*m*/*V*)) (Merck) and spread on nutrient agar medium (Merck). The soup of kombucha samples was collected and centrifuged at 1370×*g* (Mistral 2000; MSE) for 10 min. The supernatant was then filtered through a sterile filter with a pore diameter of 0.45 µm (GVS Filter Technology). Volumes of 20 µL of the filtered samples were then used to impregnate the empty disks (Bioanalyse, Ankara, Turkey). The disks were placed on inoculated plates. A volume of 20 µL of *γ*(ampicillin)=30 µg/mL (Sigma Aldrich, Merck) and 20 µL *γ*(kanamycin)=30 µg/mL (Cayman Chemical, Ann Arbor, MI, USA) were used as positive controls in the experiments. Petri dishes were incubated at 37 °C for 24 h, and the diameters of the zones of inhibition were measured. The experiments were repeated 4 times.

### Cell culture and viability assays

HCT116, a human colorectal carcinoma cell line, was cultured in RPMI 1640 medium without phenol red (Biological Industries, Beit-Haemek, Israel) supplemented with 10% fetal bovine serum (FBS; Biowest, Nuaillé, France), 1% penicillin/streptomycin (Pen/Strep; Biological Industries) and 2 mM l-glutamine (Biological Industries). The human hepatocellular carcinoma cell line Mahlavu was cultured in Dulbecco's modified Eagle medium (DMEM; Biological Industries) containing 10% FBS, 1% Pen/Strep and 1% non-essential amino acids (Biological Industries). Cells were grown in monolayers in an incubation chamber at 37 °C under a humidified atmosphere with 5% CO_2_. To determine the effects of kombucha samples on cell viability and proliferation, the 3-(4,5-dimethyl-2-thiazolyl)-2,5-diphenyl-2H-tetrazolium bromide (MTT) (Sigma-Aldrich, Merck, Taufkirchen, Germany) assay was used as described previously (*24*). Briefly, in a final volume of 100 µL of growth medium, Mahlavu and HCT116 cells were plated in microtiter plates at a density of 10^3^ and 10^4^ cell/well, respectively. The following day, the existing growth medium was replaced with a fresh medium supplemented with a kombucha beverage (0–4000 µg/mL), and the cells were incubated for 72 h. At the end of incubation, 10 µL of MTT solution, prepared at a concentration of 5 mg/mL in Dulbecco's phosphate buffered saline (Biological Industries) were added to the cells and they were incubated for an additional 4 h. Subsequently, the cells were incubated for another 24 h in the presence of 10% sodium dodecyl sulfate (SDS) in 0.01 M hydrochloric acid (HCI) and the absorbance was measured at 570 nm using a microplate spectrophotometer (Multiskan GO; Thermo Fisher Scientific, Waltham, MA, USA). All experiments were performed in triplicate with five technical replicates and cell viability (%) was calculated using the following equation:


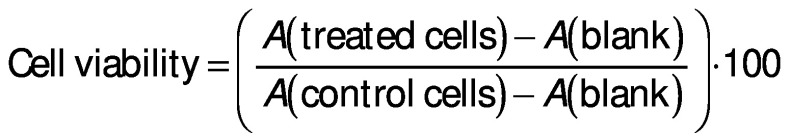
 /2/

### Sensory evaluation

Twenty individuals (12 women and 8 men) aged 19–40 were selected from the Akdeniz University campus for sensory evaluation of the samples. The participants were not informed about the contents of the beverages, since the sensory analyses were blind tests. The analysis was performed according to the techniques described by Altuğ (*25*), and the beverages were rated from 1 (very poor) to 5 (very good). Participants rated the visual and olfactory characteristics of the beverages. Sensory analysis experiments were carried out in accordance with the Declaration of Helsinki (*26*).

### Statistical analysis

Results were presented as mean value±standard deviation of three independent experiments. All statistical analyses were performed using one-way analysis of variance (ANOVA) in IBM SPSS v. 22.0 software (*27*) and Tukey's HSD and Tamhane's T2 tests. IC_50_ values were evaluated by ANOVA followed by an appropriate *post hoc* test (Duncan’s test) and differences were considered statistically significant at p<0.05. The relationship among the measured biological potentials (total phenolic content, flavonoid content and antioxidant activity) and the relationship between these biological potentials and the identified compounds were investigated by Spearman's correlation analysis (*28*).

## RESULTS AND DISCUSSION

### pH variations

After 14 days of incubation, the pH values in the herbal infusions decreased; however, the changes in the pH values of the kombucha samples were considerably more remarkable (Table 1), most likely because of the conversion of sucrose into organic acid during fermentation (*3*). Marsh *et al*. (*13*) discovered that at the end of a 10-day fermentation, the pH of kombucha cultures ranged between 3 and 3.50, which is also consistent with our results. According to Cardoso *et al*. (*29*), the pH discrepancies between fermented kombucha samples might be attributable to the predominance of distinct types of acetic acid and lactic acid bacteria. At the end of fermentation, the pH values of kombucha samples in our study varied from 2.7 to 3.6 (Table 1). According to Nummer (*30*), these values are considered safe for human consumption. The pH values above 4.2 may bring into question the microbiological safety of the fermented product and the pH values below 2.5 may be harmful to consumers due to the high concentration of organic acids.

**Table 1:** The pH values of kombucha cultures and tea infusions on days 0 and 14 of fermentation

Beverage	*t*(fermentation)/day	
0	14
pH	
Traditional kombucha	(3.8±0.0)^a^	(2.8±0.0)^*a^
Hawthorn-flavoured kombucha	(4.1±0.0)^b^	(3.1±0.1)^*b^
Hop-flavoured kombucha	(4.5±0.0)^c^	(3.6±0.3)^*c^
Madimak-flavoured kombucha	(4.3±0.0)^b,c^	(2.7±0.0)^*a^
Hawthorn tea	(6.4±0.2)^d^	(6.2±0.1)^ns,d^
Hop tea	(7.3±0.1)^e^	(7.1±0.1)^ns,e^
Madimak tea	(6.4±0.1)^d^	(6.3±0.1)^ns,d^

Results are presented as mean value±standard deviation (*N*=3). *Significant difference between day 0 and day 14 (p<0.05), ns=no significant difference between day 0 and day 14 (p>0.05). Values indicated with different letters in superscript within the same column differ significantly at p<0.05

### Profiling of microbiological composition

On day 0, the number of acetic acid bacteria, total mesophilic bacteria, yeast and *Lactobacillus* sp. ranged from 1.7·10^3^ to 7.4·10^3^ CFU/mL. After the 14-day fermentation, the number of microorganisms increased to 10^7^ in all samples, except for hawthorn-flavoured kombucha, in which the total number of mesophilic bacteria and yeasts was lower than in the other samples (5.5·10^6^ and 9.6·10^5^ CFU/mL, respectively) (Table 2).

**Table 2:** Microbiological characteristics of kombucha cultures on days 0 and 14 of fermentation

Beverage	*t*(fermentation)/day									
0		14	0	14	0		14	0	14
*N*(microorganism)/(CFU/mL)									
ACC			TMB		Yeast			*Lactobacillus*	
K	(2.3±0.4)·10^3a^	(3.0±0.3)∙10^7*a^		(6.7±0.2)∙10^3a^	(3.2±0.4)∙10^7*a^	(1.7±0.1)∙10^3a^	(2.7±0.7)∙10^7*a^		(7.4±0.3)∙10^3a^	(2.8±0.1)∙10^7*a^
KHA	(2.3±0.1)·10^3a^	(1.3±0.1)∙10^7*c^		(6.7±0.3)∙10^3a^	(5.5±0.6)∙10^6*c^	(1.7±0.3)∙10^3a^	(9.6±0.6)∙10^5*d^		(7.4±0.3)∙10^3a^	(1.6±0.1)∙10^7*d^
KH	(2.3±0.0)·10^3a^	(1.9±0.0)∙10^7*b^		(6.7±0.1)∙10^3a^	(1.3±0.4)∙10^7*b^	(1.7±0.5)∙10^3a^	(1.7±0.2)∙10^7*b^		(7.4±0.0)∙10^3a^	(2.1±0.1)∙10^7*b^
KM	(2.3±0.1)·10^3a^	(1.3±0.0)∙10^7*c^		(6.7±0.2)∙10^3a^	(1.3±0.2)∙10^7*b^	(1.7±0.2)∙10^3a^	(1.3±0.1)∙10^7*c^		(7.4±0.4)∙10^3a^	(1.9±0.1)∙10^7*c^

Results are presented as mean value±standard deviation (*N*=3). *Significant difference between day 0 and day 14 (p<0.05). Values indicated with different letters in superscript within the same column differ significantly at p<0.05. AAC=acetic acid bacteria, TMB=total mesophilic bacteria, K=traditional kombucha, KHa=hawthorn-flavoured kombucha, KH=hop-flavoured kombucha, KM=madimak-flavoured kombucha

Neffe-Skocińska *et al*. (*31*) indicated that the amount of acetic acid bacteria and yeast was around 10^4^ CFU/mL before fermentation and it increased to 10^7^ CFU/mL after 10 days of fermentation. However, Cardoso *et al*. (*29*) reached 10^5^–10^6^ CFU/mL of lactic acid bacteria, mesophilic bacteria, yeasts and acetic acid bacteria. It can be speculated that the increase in microbiological count depends on the sugar content and SCOBY composition used in the kombucha cultures.

### Bacterial cellulose production

On day 0, the concentration of SCOBY inoculated to all samples was 1.4 g/L. After 14 days of fermentation, it was found that cellulose production was the highest in the traditional kombucha ((12.8±0.7) g/L). Among the herb-flavoured kombucha samples, the highest cellulose production was observed in the fermented hawthorn tea ((10.23±0.51) g/L). The lowest cellulose production was found in the fermented hop-flavoured tea ((8.3±0.36) g/L) and fermented madimak tea ((7.9±0.46) g/L). However, there was no statistically significant difference in the amount of cellulose among the samples on day 14 (p>0.05). It is well known that cellulose production during kombucha fermentation depends on the carbon source. AL-Kalifawi and Hassan (*32*) showed that bacterial cellulose production was higher in kombucha samples prepared with 10 g/L black tea than with 5 g/L black tea. They also obtained the highest pellicle production in kombucha samples prepared with 100 g/L sucrose.

### Total phenolic and total flavonoid contents

On day 0, the highest total phenolic concentration, expressed in GAE, among the herbal kombucha samples was observed in madimak-flavoured kombucha (1469 µg/mL) (Table 3). The TPC of madimak tea (633 µg/mL) was also higher than in other tested infusions except in black tea (Table 3). After 14 days of fermentation, total phenolic concentration was the highest in madimak-flavoured kombucha samples (3151 µg/mL). Velićanski *et al*. (*33*), Shahbazi *et al*. (*7*) and Vitas *et al*. (*8*) demonstrated that the phenolic compound concentration in flavoured kombucha samples increased with fermentation. Bhattacharya *et al*. (*34*) suggested that the enzymes released by bacteria and yeast degrade polyphenols and increase the number of phenolic compounds during fermentation. On the other hand, Amarasinghe *et al*. (*35*) showed that the total phenolic concentration of the fermented kombucha samples did not increase significantly with fermentation and hypothesised that the reduction in total phenolic concentration might be related to the phenolic compounds used by the kombucha microorganisms. According to Güldane *et al*. (*4*), many factors including cultivation area, climatic conditions and the quality of agricultural practices can affect the total phenolic concentration of the tea used for fermentation. It should also be considered that the antioxidant activities of kombucha are not always determined by the total phenolic concentration, but the types of metabolites produced during fermentation may have a decisive effect (*35*, *36*).

**Table 3:** Antioxidant activity (AA), expressed in ascorbic acid equivalents (AAE), total phenolic content (TPC), expressed in gallic acid equivalents (GAE), and total flavonoids (TF), expressed in quercetin equivalents(QE), of fermented beverages on days 0 and 14 of fermentation

Beverage	*t*(fermentation)/day							
0		14	0	14	0		14
AA as *c*(AAE)/(µmol/mL)			TPC as *γ*(GAE)/(µg/mL)		TF as *γ*(QE)/(µg/mL)		
K	(625.0±1.2)^a^	(655.0±5.0)^*a^		(1262.0±2.0)^c^	(2601.0±3.0)^*b^	(34.0±0.4)^d^	(77.0±0.8)^*b^	
KHA	(548.0±3.8)^d^	(610.0±2.7)^*b^		(1354.0±3.0)^b^	(3013.0±2.0)^*a^	(38.0±0.1)^c^	(43.0±0.2)^*c^	
KH	(633.0±3.0)^a^	(634.0±1.6)^ns,a^		(1245.0±1.2)^d^	(2974.0±3.0)^*a^	(39.0±0.7)^c^	(46.0±0.3)^*c^	
KM	(559.0±3.0)^bc^	(626.0±2.6)^*a^		(1469.0±1.8)^a^	(3151.0±3.0)^*a^	(109.0±1.3)^a^	(112.0±0.2)^ns,a^	
B	(565.0±3.2)^b^	(553.0±3.4)^*c^		(1254.0±1.0)^cd^	(796.0±1.0)^*c^	(16.0±0.1)^f^	(13.0±0.2)^*e^	
HA	(209.0±1.8)^f^	(206.0±3.0)^ns,d^		(273.0±3.0)^g^	(419.0±1.8)^*d^	(11.0±0.1)^g^	(10.0±0.1)^ns,e^	
H	(234.0±2.5)^e^	(223.0±3.2)^*e^		(440.0±0.2)^f^	(526.0±3.0)^*d^	(21.0±0.2)^e^	(14.0±0.2)^*e^	
M	(554.0±3.5)^cd^	(568.0±1.4)^*c^		(633.0±1.7)^e^	(771.0±4.5)^*c^	(62.0±0.5)^b^	(65.0±0.7)^*d^	

Results are presented as mean value±standard deviation (*N*=3). *Significant difference between day 0 and day 14 (p<0.05), ns=no significant difference between day 0 and day 14 (p>0.05). Values indicated with different letters in superscript within the same column differ significantly at p<0.05. K=traditional kombucha, KHa=hawthorn-flavoured kombucha, KH=hop-flavoured kombucha, KM=madimak-flavoured kombucha, B=black tea, Ha=hawthorn, H=hop, M=madimak

It was found that the total flavonoid concentration of traditional kombucha, hawthorn-flavoured kombucha and hop-flavoured kombucha samples increased significantly with fermentation (p<0.05) (Table 3). At the end of the fermentation, the traditional kombucha had a total flavonoid concentration, expressed in QE, of 77 µg/mL, the hawthorn-flavoured kombucha of 43 µg/mL and the hop-flavoured kombucha of 46 µg/mL. Although the highest concentration of total flavonoids was found in the madimak-flavoured kombucha beverage (109 µg/mL) before fermentation, no significant difference was found between the unfermented and fermented madimak-flavoured kombucha samples (p>0.05). Similarly, madimak had the highest total flavonoid concentration among the teas (Table 3). There was also a moderate correlation between the total phenolic and flavonoid concentration of the samples (r=0.58, p<0.05). In their study, Vitas *et al*. (*8*) prepared alternative kombucha drinks from six medicinal plants, namely winter savoury, peppermint, nettle, wild thyme, elderberry and quince. They showed that alternative kombucha drinks prepared with winter savoury, peppermint and nettle had higher total flavonoid concentration than conventional products. In the same study, they found that the total flavonoid concentration of kombucha samples prepared with quince decreased with fermentation. Shahbazi *et al*. (7) also demonstrated that the flavonoid concentration of the kombucha samples fermented with medicinal herbs increased significantly. According to the researchers, this increase could be explained by the production of catechin and epicatechin isomers.

### LC-MS analysis of phenolic compounds

After fermentation, gallic acid was the major phenolic compound in all kombucha samples, except for the madimak-flavoured kombucha (Table 4). However, chlorogenic acid was present at the highest concentration in madimak-flavoured kombucha. Traditional kombucha had a higher content of caffeic acid and vitexin than other samples. On the other hand, hawthorn-flavoured kombucha had a higher concentration of epicatechin. Rutin and protocatechuic acid contents did not significantly differ among the beverages (p>0.05). Some phenolic acids such as caffeic acid, rutin, protocatechuic acid and vitexin are known to be antioxidant, antibacterial, antimutagenic, and anticarcinogenic agents (*37*). Gallic acid is important in terms of its anti-obesity properties (*e.g.* enhancement of insulin signalling, or suppression of lipogenesis), as well as for anticancer, gastrointestinal mucosal protection and antibacterial properties (*37*).

**Table 4:** The concentration of polyphenols in kombucha samples after 14 days of fermentation

Polyphenol	*γ*(polyphenol)/(mg/mL)				
K	KHa	KH		KM
Caffeic acid	(84.9±0.9)^a^	(26.5±1.2)^b^		(1.8±0.1)^c^	(15.0±2.4)^bc^
Chlorogenic acid	(26.0±0.4)^c^	(103.0±2.3)^b^		(15.5±0.7)^c^	(868.4±6.8)^a^
Epicatechin	(32.9±0.5)^b^	(51.1±4.6)^a^		(32.9±1.5)^b^	(14.3±0.2)^c^
Gallic acid	(666.2±19.5)^b^	(879.1±5.8)^a^		(432.5±10.7)^d^	(572.1±14.7)^c^
Protocatechuic acid	nd	(0.6±0.0)^a^		(0.2±0.0)^a^	(1.1±0.0)^a^
Rutin	(56.0±0.8)^a^	(42.1±1.2)^a^		(41.7±0.8)^a^	(40.2±0.7)^a^
Vitexin	(80.3±3.3)^a^	(68.3±1.7)^ab^		(55.3±1.4)^b^	(74.5±0.8)^a^

Results are presented as mean value±standard deviation (*N*=3). Different letters in superscript within the same row indicate significant difference of samples on day 14 (p<0.05). K=traditional kombucha, KHa=hawthorn-flavoured kombucha, KH=hop-flavoured kombucha, KM=madimak-flavoured kombucha, nd=not detected

**Fig. S1:**
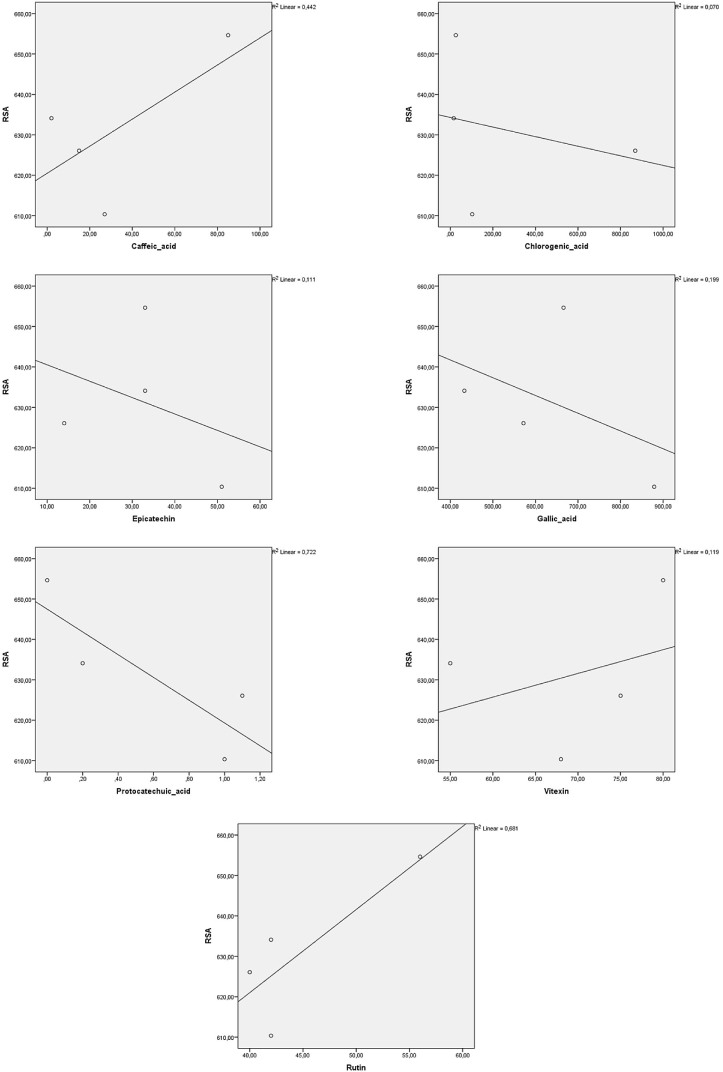
Spearman’s correlation between the identified compounds and the measured free radical scavenging activity (RSA)

**Fig. S2:**
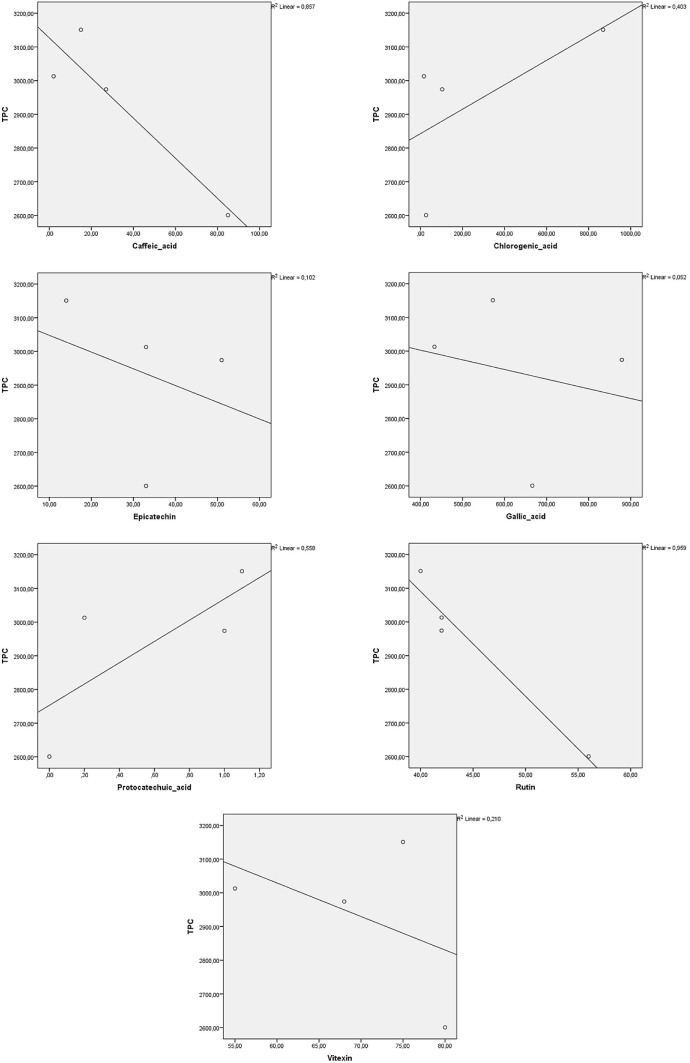
Spearman’s correlation between the identified compounds and the measured total phenolic content (TPC)

**Fig. S3:**
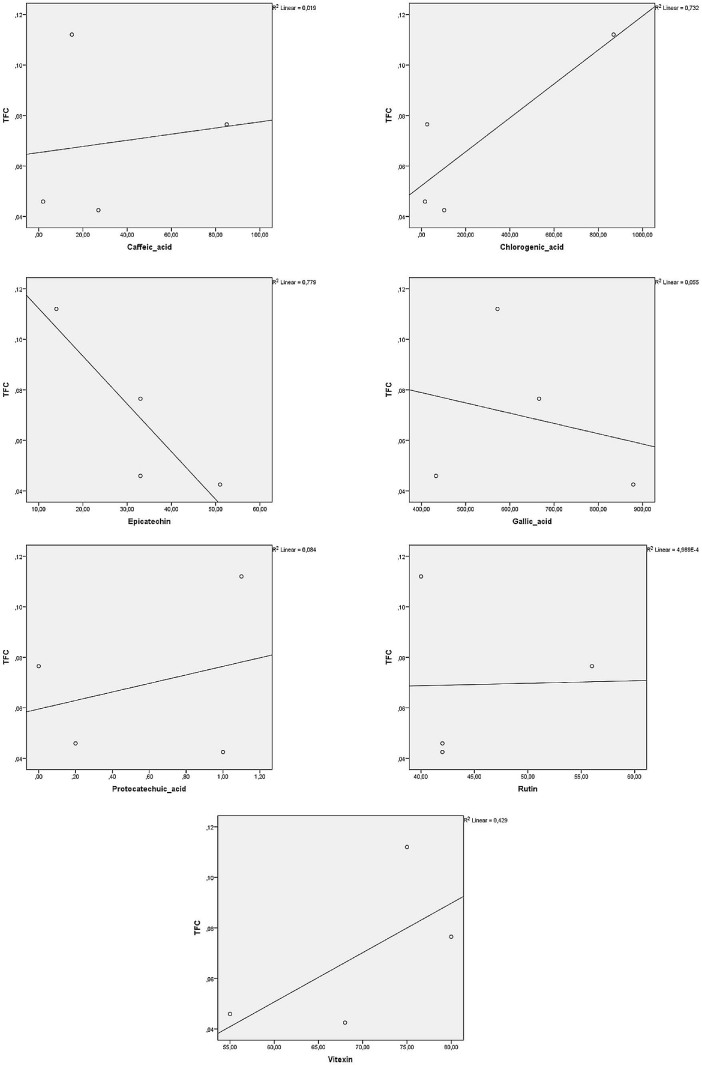
Spearman’s correlation between the identified compounds and the measured total flavonoid content (TFC)

The results showed that caffeic acid and rutin are positively correlated with antioxidant capacity (Fig. S1), but negatively correlated with phenolic and flavonoid concentration (Fig. S2 and Fig. S3, respectively). On the contrary, chlorogenic acid and protocatechuic acid correlated negatively with antioxidant capacity (Fig. S1) and positively with phenolic and flavonoid concentration (Fig. S2 and Fig. S3, respectively). Vitexin showed a negative correlation with phenolic concentration (Fig. S2), whereas it showed a positive correlation with the antioxidant and flavonoid concentration (Fig. S1 and Fig. S3, respectively). Interestingly, epicatechin and gallic acid were found to be negatively correlated with all three biological potentials. As is well known, the phenolic compounds identified in this study by the LC analysis are only a few of the many substances found in kombucha. Also, it is quite possible that these substances work in concert with one another. As a result, the direct positive correlation effect of the mentioned phenolic compounds on the biological activities of kombucha may not always be clear. This study may not have identified the phenolic compounds in kombucha that correlate strongly with other biological potentials.

In their study, Borges *et al*. (*38*) pointed out the relationship between gallic acid and antimicrobial activity, cell surface hydrophobicity, K^+^ leakage, charge and induced propidium iodide (PI) uptake. Chlorogenic acid is one of the most available and naturally occurring phenolic acids in tea and green coffee (*39*). The pronounced radical scavenging activity of chlorogenic acid and its polyphenol degradation products was already demonstrated by Bøhn *et al*. (*40*) and was significantly high in madimak-flavoured kombucha beverages in our study. Chlorogenic acid is known to have antimicrobial activity, but it is not effective against Gram-positive lactic acid bacteria, making it a food additive and preservative (*41*). The presence of chlorogenic acid in madimak-flavoured kombucha not only explains the high antibacterial activity of the beverage, but also extends its shelf life potential due to the food preservative properties of the acid.

### Free radical scavenging activity

The traditional kombucha beverage fermented for 14 days had the highest antioxidant activity (655 µmol/mL) among all samples, followed by hop-flavoured kombucha (634 µmol/mL) (Table 3). It was found that the antioxidant activity of traditional kombucha, hawthorn-flavoured kombucha and madimak-flavoured kombucha increased significantly with fermentation (p<0.05). There was a significant difference between the antioxidant activities, expressed in AAE, of the unfermented hawthorn-flavoured kombucha and fermented hawthorn-flavoured kombucha (548 and 610 µM/mL, respectively) (p<0.05). Similarly, the madimak-flavoured kombucha and the traditional kombucha showed higher antioxidant activity (626 and 655 µmol/mL, respectively) than their unfermented controls. Moreover, the antioxidant activity of the madimak tea (568 µmol/mL) and black tea (553 µmol/mL) was significantly higher than that of all herbal infusions after 14 days (Table 3). In general, it can be concluded that the presence of black tea in kombucha cultures is associated with the increased radical scavenging activity. In addition, it was found that there was a strong correlation between the increase in total flavonoid concentration and the increase in the antioxidant activity, and this correlation was statistically significant (r=0.69, p<0.05). However, the results of antioxidant capacity and total phenolic concentration showed that there was a weak correlation between these two tests, which was not statistically significant (r=0.49, p>0.05).

Chu and Chen (*36*) showed that the DPPH activity of various Taiwanese household kombucha samples increased with fermentation. However, on the 10th day of fermentation, the increase was significant only in some samples. The researchers concluded that the differences in the microbiota of the kombucha samples were responsible for the different radical scavenging activities. Tanticharakunsiri *et al*. (*42*) showed that radical scavenging activities of oolong tea and kitchen mint kombucha samples increased during fermentation and had the highest antioxidant capacity on day 14 of fermentation. Shahbazi *et al*. (*7*) demonstrated that the radical scavenging activity, total phenolic and flavonoid concentration of kombucha samples flavoured with Shirazi thyme, cinnamon and cardamom increased with 16 days of fermentation. However, Vitas *et al*. (*8*) showed that the antioxidant activity of winter savory-, green tea- and stinging nettle-flavoured kombuchas increased until the third day of fermentation and then decreased. In addition, the antioxidant activity of wild thyme-, elderberry-, quince- and peppermint-flavoured kombuchas was high at the beginning of fermentation and decreased continuously with fermentation (*8*). It has already been shown that the microbial composition of the SCOBY has a great effect on the resulting beverage (*35*). It is known that the higher antioxidant activity of fermented kombucha than of its unfermented form is due to the bacteria and yeast enzymes causing structural changes in the polyphenols in the tea and the production of low molecular mass components. The antioxidant activities in kombucha cultures show time-dependent changes and a long fermentation time is not recommended due to the harmful accumulation of organic acids (*43*). Therefore, the fermentation duration was limited to 14 days in this study.

### Antibacterial activity

In order to evaluate the antibacterial activity of the samples, the inhibition zones around the sample disks (in mm) were measured (Table 5). None of the kombucha beverages showed any antibacterial activity on day 0. After 14 days of fermentation, the traditional kombucha showed antibacterial activity against *K. pneumoniae*, *B. cereus*, *S. epidermidis* and *E. coli*, and the madimak-flavoured kombucha showed antibacterial activity against all six bacterial strains. Furthermore, madimak-flavoured kombucha formed a larger zone of inhibition against *P. aeruginosa* than kanamycin (p<0.05). Hop-flavoured kombucha showed antibacterial activity against *K. pneumonia, B. cereus*, *P. aeruginosa, S. epidermidis* and *E.* *coli*, and hawthorn-flavoured kombucha showed antibacterial activity against *K. pneumonia, S. aureus*, *B. cereus*, *S. epidermidis* and *E. coli*. In contrast to ampicillin, which had no antibacterial activity against *E. coli*, all the herb-flavoured kombucha samples were effective against *E. coli* (p<0.05). Besides, all flavoured kombucha samples formed a larger zone of inhibition than ampicillin against *K. pneumonia* (p>0.05).

**Table 5:** Average diameter of inhibition zones of kombucha samples fermented for 14 days

Sample	*d*(inhibition zone)/mm												
KP	SA		BC		PA		SE		EC			
Ampicillin	(7.0±0.0)^bc^		(24.5±1.9)^a^		(11.0±1.4)^b^		(10.5±1.0)^ab^		(17.5±1.0)^a^			(6.0±0.0)^d^		
Kanamycin	(17.0±1.2)^a^		(16.0±0.7)^b^		(16.0±1.1)^a^		(8.0±0.0)^cd^		(17.0±1.4)^a^			(16.5±1.0)^a^		
K	(8.0±0.2)^bc^		(6.0±0.0)^d^		(8.0±0.8)^cd^		(6.0±0.0)^d^		(8.0±0.6)^c^				(8.0±0.0)^cd^		
KHa	(9.0±1.4)^b^		(9.0±1.4)^c^		(8.0±0.4)^cd^		(6.0±0.0)^d^		(8.0±1.1)^c^			(9.0±1.2)^c^		
KH	(8.0±0.2)^bc^		(6.0±0.0)^d^		(10.1±0.4)^bc^		(10.0±0.2)^bc^		(8.0±0.1)^c^			(10.0±0.0)^bc^		
KM	(8.5±1.0)^b^		(9.0±1.4)^c^		(12.0±0.0)^b^		(12.5±1.0)^a^		(10.5±1.0)^b^			(12.0±0.0)^b^		
B	(6.0±0.0)^c^		(6.0±0.0)^d^		(6.0±0.0)^d^		(6.0±0.0)^d^		(6.0±0.0)^c^			(6.0±0.0)^d^		
Ha	(6.0±0.0)^c^		(6.0±0.0)^d^		(6.0±0.0)^d^		(6.0±0.0)^d^		(6.0±0.0)^c^			(6.0±0.0)^d^		
H	(6.0±0.0)^c^		(6.0±0.0)^d^		(6.0±0.0)^d^		(6.0±0.0)^d^		(6.0±0.0)^c^			(6.0±0.0)^d^		
M	(6.0±0.0)^c^		(6.0±0.0)^d^		(6.0±0.0)^d^		(6.0±0.0)^d^		(6.0±0.0)^c^		(6.0±0.0)^d^		

Results are presented as mean value±standard deviation (*N*=3). Different letters in superscript within the same column indicate the significant difference of samples on day 14 (p<0.05). The discs used in the study have a *d*=6 mm. A value of 6 in the table indicates that no inhibition zone is formed. BC=*Bacillus cereus*, SA=*Staphylococcus aureus*, SE=*Staphylococcus epidermidis*, EC= *Escherichia coli*, KP=*Klebsiella pneumonia*, PA*=Pseudomonas aeruginosa*. K=traditional kombucha, KHa=hawthorn-flavoured kombucha, KH=hop-flavoured kombucha, KM=madimak-flavoured kombucha, B=black tea, Ha=hawthorn tea, H=hop tea, M=madimak tea

Sreeramulu *et al*. (*3*) observed that unfermented black tea and traditional kombucha did not have antibacterial activity against bacterial strains except *Campylobacter jejuni*. Previously, black tea kombucha fermented for 10 days was shown to inhibit the growth of *S. aureus* and *Listeria monocytogenes* (minimum inhibitory concentration 250 µL/mL), whereas it had no antibacterial activity against *E. coli* and *Salmonella* (*29*). These differences could be due to the influence of different parameters in kombucha fermentation, such as the origin of the SCOBY, the amount of soup, fermentation time, sugar concentration and temperature, as these factors may lead to the formation of antibacterial compounds (bacteriocins, organic acids, enzymes, proteins, *etc*.) at different concentrations (*29*).

### Cytotoxic effects of kombucha beverages on cancer cells

Antiproliferative potential of traditional kombucha and kombucha beverages flavoured with different medicinal plants was investigated in two different cancer cell lines, HCT116 and Mahlavu. The efficacy of the kombucha beverages was assessed by measuring the percentage of surviving cells after 72 h of incubation with different concentrations of the test compounds. The IC_50_ values of kombucha beverages are given in Table 6. The results indicate that Mahlavu cells are generally more resistant to the cytotoxic effect of kombucha beverages than HCT116 cells. The only exception to this generalisation was hop-flavoured kombucha, which had a higher cytotoxic effect on Mahlavu cells.

**Table 6:** *In vitro* cytotoxic effects of kombucha beverages against human colorectal carcinoma HCT116 and human hepatocellular carcinoma Mahlavu

Beverage	IC_50_/(mg/mL)	
HCT116	Mahlavu
Traditional kombucha	(0.8±0.1)^bc^	(1.5±0.3)*^a^
Madimak-flavoured kombucha	(0.6±0.0)^c^	(1.1±0.1)*^b^
Hop-flavoured kombucha	(0.8±0.0)^bc^	(0.7±0.2)^ns,c^
Hawthorn-flavoured kombucha	(0.9±0.1)^a^	(1.3±0.1)*^ab^

Results are presented as mean value±standard deviation (*N*=3). *Statistically significant difference in the same row (p<0.05), ns=no significant difference in the same row (p>0.05). Values indicated with different letters in superscript differ significantly within same column at p<0.05. IC_50_=half maximal inhibitory concentration

**Fig. 1:**
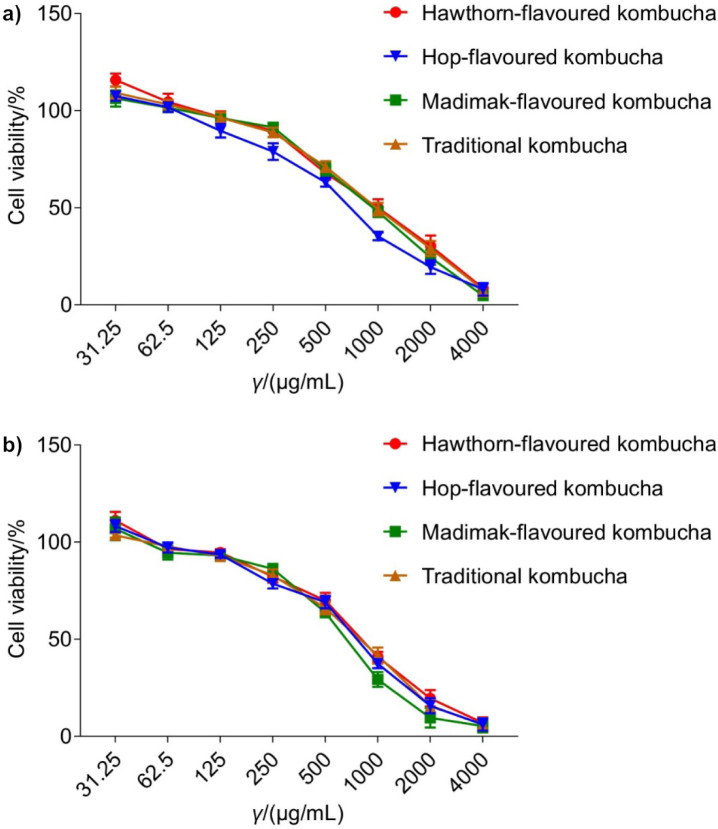
Effects of various concentrations of different kombucha beverages on the viability of: a) Mahlavu and b) HCT116 cells

Fig. 1 also shows the effects of the tested kombucha beverages on cell viability in a dose-dependent manner. As it can be seen, extremely low concentrations of kombucha drinks (*i.e.* 31.25 µg/mL) slightly enhanced cell viability, while higher concentrations of kombucha beverages had dose-dependent cytotoxic effects on Mahlavu and HCT116 cells. Cancer is known to be one of the leading causes of death worldwide (*44*), and this fact underscores the importance of research to develop novel molecules/drugs with anticancer properties. Much research in recent years has focused on the anticancer properties of kombucha as a functional beverage (*45*, *46*). It is known that the anticancer properties of kombucha vary depending on the parameters such as the content of microorganisms in the symbiotic culture used for kombucha fermentation, fermentation time, sucrose content, temperature, type of tea, and the presence of herbal infusions (*47*). Herein, we investigated the growth inhibitory potential of traditional kombucha and kombucha beverages flavoured with hop, madimak and hawthorn on the hepatocellular carcinoma cell line Mahlavu and the colorectal carcinoma cell line HCT116. The results revealed that each kombucha beverage had a strong cytotoxic effect on both cell lines. Madimak-flavoured kombucha proved to be more effective on HCT116 cells, while hop-flavoured kombucha had a stronger cytotoxic effect on Mahlavu cells. These antiproliferative effects of kombucha beverages could be due to the polyphenols or secondary metabolites produced during the fermentation (*48*). Nevertheless, further studies are needed of the toxicity of kombucha on various cancer cell lines and normal cell lines. A recent study also revealed that the anticancer effect of doxorubicin on cancer cells increased in the presence of kombucha (*49*). In this context, it would be beneficial to investigate the combinatorial use of different chemotherapeutic agents together with kombucha fermented in the presence/absence of various herbal infusions.

### Hedonic ratings of sensory evaluation

**Fig. 2:**
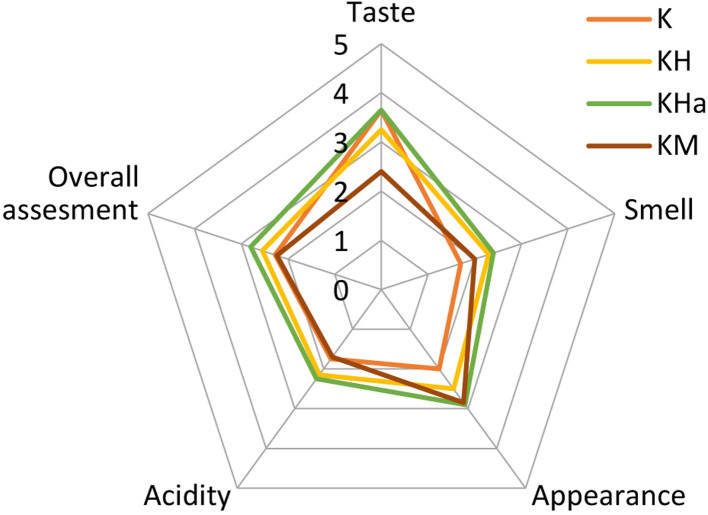
Sensory analysis results of 14-day-old kombucha samples. K=traditional kombucha, KH=hop-flavoured kombucha, KHa=hawthorn-flavoured kombucha, KM=madimak-flavoured kombucha

Participants rated the appearance, odour, acidity, taste and overall quality of kombucha beverages after 14 days of fermentation on a 5-point hedonic scale (Fig. 2). The participants stated that the produced fermented beverages were generally blurry and had a pungent odour and taste. Traditional kombucha and hawthorn-flavoured kombucha were rated as the tastiest of all beverages (3.7). The odour of the kombucha samples was generally disliked by the participants. The odour (2.4) and appearance (2.9) of the hawthorn-flavoured kombucha received the best scoring among the samples. In general, the hawthorn-flavoured kombucha was perceived as the most pleasant drink according to the results of the sensory analysis (2.8). Ulusoy and Tamer (*50*) indicated that a shorter fermentation time leads to a slight decrease in the sugar content of the beverage, making subjects more likely to enjoy the taste of the beverage. The taste of the drink is likely to be appreciated by the participants because a short fermentation time prevents the formation of a vinegar taste caused by organic acids. Therefore, it is probable that the 14-day fermentation period was what led to the low taste rating of the beverages in our study.

## CONCLUSIONS

The effects of three medicinal plants, namely hawthorn, hop and madimak on the antimicrobial and antiproliferative properties, bioactivity, microbial profile, sensory properties and cellulose production of kombucha beverage were examined in this study. Although the antioxidant activities and the total concentration of phenolic substances in the three alternative kombucha beverages were similar, the hop-flavoured kombucha showed a strong cytotoxic effect against Mahlavu cells. Hawthorn-flavoured kombucha was rated as the most delicious drink by the participants. Madimak-flavoured kombucha also appears to be a promising product among kombucha beverages due to its strong antibacterial activity and high phenolic and flavonoid concentrations. It also had a strong cytotoxic effect against HCT116 cell line. However, the results of the sensory analysis indicate that the sensory characteristics of all herb-flavoured kombucha beverages need to be improved to appeal to consumers. For instance, increasing the gluconic acid ratio of the beverages, determining the sugar, alcohol and organic acid content, and determining the conditions of the fermentation process and their effects on sensory properties may be effective in improving the flavour quality and control of kombucha. In addition, the effects of these kombucha products on model organisms can be studied and their health-promoting effects commented on.
